# Expanding geographic distribution knowledge of *Galerinamarginata* (Batsch) Kühner (Agaricales, Hymenogastraceae) with a novel Antarctic record

**DOI:** 10.3897/BDJ.12.e125727

**Published:** 2024-06-21

**Authors:** Fernando Augusto Bertazzo-Silva, Jair Putzke, Cassiane Furlan-Lopes, Maricia Fantinel D'ávila, Alice Lemos Costa, Evelise Leis Carvalho, Ana Flavia Zorzi, Carlos Ernesto Gonçalves Reynaud Schaefer

**Affiliations:** 1 Laboratório de Taxonomia de Fungos, Universidade Federal do Pampa (UNIPAMPA), São Gabriel, RS, Brazil Laboratório de Taxonomia de Fungos, Universidade Federal do Pampa (UNIPAMPA) São Gabriel, RS Brazil; 2 Universidade Federal do Pampa (UNIPAMPA), São Gabriel, RS, Brazil Universidade Federal do Pampa (UNIPAMPA) São Gabriel, RS Brazil; 3 Universidade Federal de Viçosa, Departamento de Solos, Viçosa, MG, Brazil Universidade Federal de Viçosa, Departamento de Solos Viçosa, MG Brazil

## Abstract

**Background:**

The investigation of Agaricales diversity in the Antarctica is limited, with only seven genera reported for the region. *Galerina* stands out as the genus with the highest species diversity, including 12 species in Antarctica. This research reports the presence of *G.marginata* in the region, providing the first complete morphological description for the specimen developing in Antarctica. Sampling was conducted during the Austral summer of 2022/2023 as part of the XLI Brazilian Antarctic Operation in Point Smellie, Byers Peninsula, Livingston Island, South Shetland Archipelago, Antarctica. Phylogenetic relationships reconstructed by Maximum Likelihood demonstrate that *G.marginata* forms a monophyletic clade with over 60% bootstrap support in most branches. The isolate in this study was found to be internal to the main cluster. Evolutionary reconstructions using the Maximum Likelihood method indicate that the branches correspond to the Antarctic isolate being an internal clade within the *marginata* group. Recording fungal populations in polar regions offers information about their adaptation and survival in inhospitable environments. Understanding the species' distribution in Antarctica encourages future investigations into its ecology and interactions with other organisms. Here, data are presented to establish an initial foundation for monitoring the *G.marginata* population in Antarctica and assessing the potential impacts of climate change on its development and survival in the forthcoming years.

**New information:**

We report the third occurrence of *Galerinamarginata* (Batsch) Kühner in Antarctica and provide, for the first time, a comprehensive morphological description of an individual of the species for the Antarctic continent, accompanied by phylogenetic analyses and comprehensive discussions regarding its diversity and global distribution.

## Introduction

The Antarctic continent is known as one of the most inhospitable places on the Planet due to its meteorological conditions and geographical isolation (Lecordier et al. 2023). It is divided into two primary sectors: Continental Antarctica, which includes ice-free regions such as the McMurdo Dry Valleys, Windmill Islands and inland nunataks; and Maritime Antarctica, which extends along the western coast of the Antarctic Peninsula and the archipelagos of the Scotia Arc, reaching northwards to South Georgia, which is designated as part of the sub-Antarctic region (Colesie et al. 2023).

The Antarctic Peninsula and adjacent islands in Maritime Antarctica experience distinct seasons influenced by the Southern Ocean. Summers are brief, with temperatures above freezing and extended daylight hours, significantly impacting annual precipitation. Conversely, winters are characterised by temperatures ranging from -10°C to -12°C, prolonged nights and the formation of sea ice (Yu et al. 2024). In Polar Regions, physical environmental conditions often exert a more pronounced influence than biological factors (Peck et al. 2006). This suggests that climate, soil, ice and other physical factors play a substantial role, often overriding biological conditions in determining the life and adaptation of organisms in these regions.

Due to the extreme conditions in Antarctica, research on its biodiversity is crucial for understanding the distribution and adaptive capabilities of extremophile organisms. Although more than 70 genera of Agaricales have been reported for the continent in biodiversity databases (GBIF.org 2024b), the diversity and distribution of Agaricales in Antarctica in scientific literature remain understudied. Reports indicate only seven genera in the region: *Arrhenia* Fr. (= *Leptoglossum* P. Karst.), *Galerina* Earle, *Lichenomphalia* Redhead, Lutzoni, Moncalvo and Vilgalys (= *Owingsia* I. Saar, Voitk and Thorn), *Omphalina* Quél., Pholiota (Fr.) P. Kumm., *Rimbachia* Pat. and *Simocybe* P. Karsten (Putzke et al. 2012, Palfner et al. 2020, MycoBank Database 2024). Amongst these, *Galerina* stands out as the genus with the highest species diversity, with 12 species thriving in the Antarctic Region, as indicated by Garrido-Benavent et al. (2023).

The genus *Galerina* comprises approximately 300 species and typically produces basidiomata commonly associated with living bryophytes, functioning as saprophytes or being confined to woody material and other plant remnants (Horak 1994, Gulden et al. 2005). Recent studies have shown that lethal mushrooms of the genus colonised Antarctica as early as the Pleistocene, concurrent with the estimated colonisation of plants such as the grass *Deschampsiaantarctica* Desv., mosses and some amphitropical lichens on the continent (Garrido-Benavent et al. 2023). Despite their presence on the Antarctic continent for thousands of years, studies related to the genus *Galerina* are still in their early stages, highlighting the need for more comprehensive research on their diversity in Antarctica to clarify distribution issues and provide consistent information on the impacts of climate change on *Galerina* spp. populations.

This study presents a novel finding of *Galerinamarginata* (Batsch) Kühner in Antarctica through the collection of Agaricales fungi in the South Shetland Islands. The primary contribution of this research lies in providing new insights into the morphology, phylogeny and distribution of *G.marginata* on the Antarctic Continent, offering novel and relevant information on the bionomy and ecology of this species in the region. This research aims to report, for the first time in scientific literature, the presence of *G.marginata* on the Byers Peninsula (Livingston Island, Antarctica), providing morphological and phylogenetic data on the species.

## Materials and methods

### Study site

**Figure 1.**
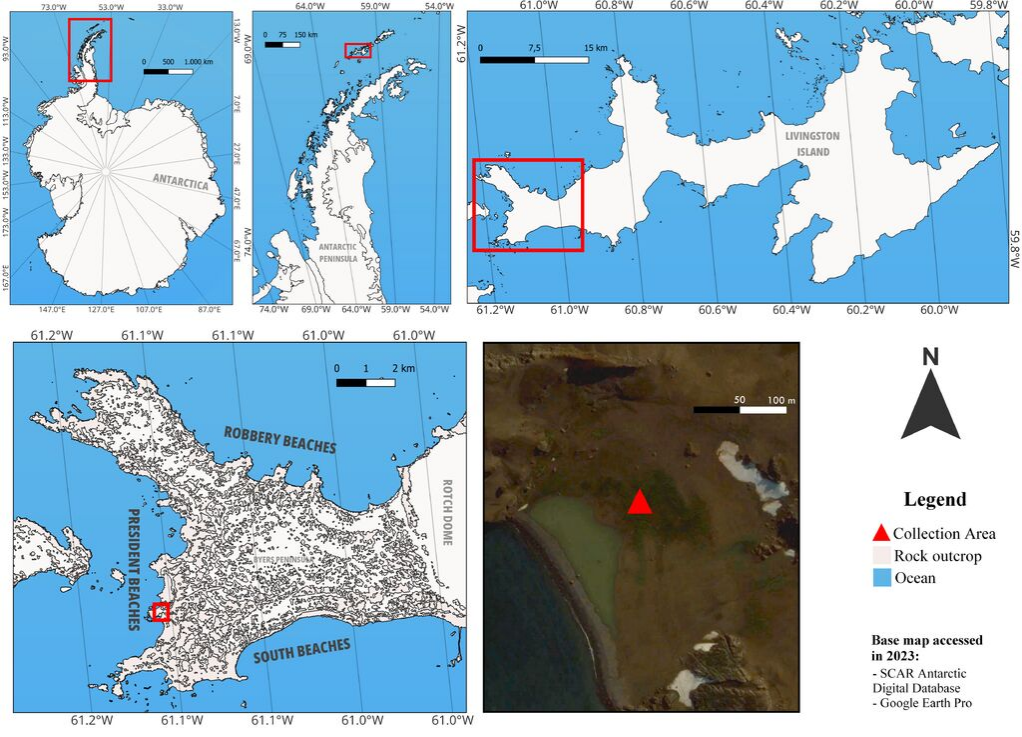
Location map of the collection area. Antarctica, Antarctic Peninsula, Livingston Island, Byers Peninsula, Point Smellie. Created in QGIS 3.32.0 software and designed in PhotoFiltre Studio X 10.12.1 software.

The samplings were carried out in the austral summer of 2022/2023 as part of the XLI Brazilian Antarctic Operation at Point Smellie, Byers Peninsula, Livingston Island, South Shetland Archipelago, Antarctica (62°39'12.6"S, 61°08'44.6"W) (Fig. 1). The specimens were photographed and collected using tweezers, along with detailed documentation of the mushrooms' morphology, the geographical coordinates of their location and the substrate on which they were discovered. Subsequently, a fraction of the specimens underwent desiccation in a 40°C oven, while the remaining portion was preserved fresh for molecular analysis. All samples were transported to Brazil and stored frozen at -20°C.

### Morphological characterisation and classification

The morphological characterisation and examination of organism structures were initially conducted in the field in Antarctica and subsequently continued at the Laboratório de Taxonomia de Fungos (LATAF) at Universidade Federal do Pampa (UNIPAMPA), São Gabriel, Rio Grande do Sul State, Brazil. Microscope slides were prepared using a 3% potassium hydroxide (KOH) solution and observed under the Axio Scope A1 Binocular Microscope. The microscopic characterisation involved measuring 25 specimens of each microscopic structure, with means followed by standard deviations presented and the maximum and minimum sizes of each structure included in parentheses. Following the analyses, samples of the material were dehydrated in an oven at 40°C and subsequently deposited in the Bruno Edgar Irgang Herbarium (HBEI 127).

For the verification of the current taxonomic classification and synonyms of *Galerinamarginata*, the Catalogue of Life and Index Fungorum platforms were employed. To analyse the worldwide geographical distribution of the species, specialised bibliographies, the Global Biodiversity Information Facility and Barcode of Life Data System platforms were consulted.

### DNA extraction, PCR amplification and sequencing

Fungal DNA extraction utilised desiccated frozen tissue obtained from *G.marginata*. These extractions were performed using an E.Z.N.A.® Fungal DNA Mini Kit, Omega Bio-tek. DNA sequences of the ITS region (ITS1–5.8S–ITS2) were obtained by primers ITS1 (5’-CTTGGTCATTTAGAGGAAGTAA-3’) and ITS4 (5’-TCCTCCGCTTATTGATATGC-3’) (Sappington and Taylor 1990, White et al. 1990). PCR was performed for a final volume of 25 μl containing: 25 ng of genomic DNA (1 μl), 20 mM of each primer (0.25 μl) 10 mM of dNTP mix (2 μl), 50 mM of MgCl_2_ (0.75 μl) 10 × PCR buffer (2.5 μl), Taq polymerase at 5 U/μl (0.25 μl) (Ludwig Biotecnologia) and Milli-Q water to complete the reaction. The PCR reaction was performed following White et al. (1990) adapted conditions: 94°C for 2 min, followed by 35 cycles at 94°C for 30 s, 55°C for 40 s and 72°C for 1 min and a final extension of 72°C for 1 min. The PCR product was purified using a column PCR Product Purification Kit (Ludwig Biotecnologia) and sequenced automatically in a sequencer (ABI 3500 XL Applied Biosystems).

### Phylogenetic analysis

For the phylogenetic analysis, the BLAST search was performed at the National Center for Biotechnology Information (NCBI) and closely related sequences were downloaded from GenBank. Evolutionary analysis using the Maximum Likelihood method was conducted to reconstruct the evolutionary history of *G.marginata*. The Tamura 3-parameter model (Tamura and Nei 1993) was employed within the Maximum Likelihood framework. A bootstrap consensus tree was inferred from 10,000 replicates (Kumar et al. 2018). Branches in partitions reproduced in fewer than 50% of bootstrap replicates were collapsed (Felsenstein 1985). The percentage of replicate trees in which the associated taxa clustered together in the bootstrap test (10,000 replicates) is shown next to the branches. Initial trees for the heuristic search were automatically obtained by applying the Neighbour-Joining and BioNJ algorithms to a matrix of pairwise distances estimated using the Maximum Composite Likelihood (MCL) approach, then selecting the topology with the highest log likelihood value. A discrete Gamma distribution was utilised to model evolutionary rate differences amongst sites (1,000 categories; G parameter = 0.2683). The analysis involved 51 taxa (nucleotide sequences), with 47 in the in-group and four in the out-group. (Table 1). There were a total of 1,303 positions included (1st + 2nd + 3rd + non-coding) in the final dataset. Evolutionary analyses were conducted using RAxML v.8 (Stamatakis 2016).

## Taxon treatments

### Galerina marginata

(Batsch) Kühner

57129C60-DA00-53E0-8659-9A2FE5453A1F

253217

= *Agaricusautumnalis* Peck, Ann. Rep. Reg. N.Y. St. Mus. 23: 92 (1872) [1870]
= Agaricuscaudicinusvar.denudatus Pers., Syn. meth. fung. (Göttingen) 2: 272 (1801)
= *Agaricusmarginatus* Batsch, Elench. fung., cont. sec. (Halle): 207 (1789)
= Agaricusmutabilisvar.marginatus (Batsch) Fr., Hymenomyc. eur. (Upsaliae): 225 (1874)
= *Agaricusunicolor* Vahl, Fl. Danic. 6: 7 (1792)
= *Dryophilamarginata* (Batsch) Quél., Enchir. fung. (Paris): 69 (1886)
= *Dryophilaunicolor* (Vahl) Quél., Enchir. fung. (Paris): 69 (1886)
= *Flammulamarginata* (Batsch) Fayod, Annls Sci. Nat., Bot., sér. 7 9: 361 (1889)
= *Galeramarginata* (Batsch) P. Kumm., Führ. Pilzk. (Zerbst): 74 (1871)
= *Galeraunicolor* (Vahl) Ricken, Die Blätterpilze: Pl. 56, figs 4, 7 (1912)
= *Galerinaautumnalis* (Peck) A.H. Sm. and Singer, Monogr. Galerina: 246 (1964)
= Galerinaautumnalisf.robusta Thiers, Mycologia 51(4): 534 (1960) [1959]
= Galerinaautumnalisvar.angusticystis A.H. Sm., Monogr. Galerina: 249 (1964)
= Galerinaautumnalisvar.robusta Thiers, Beitr. Naturk. Forsch. Südwestdeutschl.: 249 (1964)
= *Galerinaunicolor* (Vahl) Singer, Beih. Botan. Centralbl., Abt. 2 56: 170 (1936)
= Galerinaunicolorf.fibrillosa Arnolds, Biblthca Mycol. 90: 379 (1982)
= Galerinaunicolorf.paucicystidiata Arnolds, Biblthca Mycol. 90: 378 (1982)
= *Galerulamarginata* (Batsch) Kühner, Bull. trimest. Soc. mycol. Fr. 50: 78 (1934)
= *Galerulaunicolor* (Vahl) Kühner, Bull. trimest. Soc. mycol. Fr. 50: 78 (1934)
= *Gymnopilusautumnalis* (Peck) Murrill, N. Amer. Fl. (New York) 10(3): 200 (1917)
= *Naucoriaautumnalis* (Peck) Sacc., Syll. fung. (Abellini) 5: 834 (1887)
= *Pholiotaautumnalis* (Peck) Peck, Bull. N.Y. St. Mus. 122: 156 (1908)
= *Pholiotamarginata* (Batsch) Quél., Mém. Soc. Émul. Montbéliard, Sér. 2 5: 127 (1872)
= Pholiotamarginatasubsp.mustelina (Quél.) P. Karst., Bidr. Känn. Finl. Nat. Folk 32: 305 (1879)
= Pholiotamarginatavar.tremulae Pilát, Stud. Bot. Čechoslov. 11: 166 (1950)
= *Pholiotaunicolor* (Vahl) Gillet, Hyménomycètes (Alençon): 436 (1876) [1878]
= *Ryssosporamarginata* (Batsch) Fayod, Annls Sci. Nat., Bot., sér. 7 9: 361 (1889)

#### Materials

- **Type status:** Other material. **Occurrence:** associatedSequences: GenBank: PP346498.1; occurrenceID: 7209E6AB-A5FE-5819-A500-5CE116BBC10D; **Taxon:** scientificNameID: *Galerinamarginata*; kingdom: Fungi; phylum: Basidiomycota; class: Agaricomycetes; order: Agaricales; family: Hymenogastraceae; genus: Galerina; specificEpithet: *marginata*; scientificNameAuthorship: (Batsch) Kühner; taxonomicStatus: accepted; **Location:** continent: Antarctica; islandGroup: South Shetland Islands; island: Livingston Island; locality: Byers Peninsula, Point Smellie.; verbatimLatitude: 62°39'12.6"S; verbatimLongitude: 61°08'44.6"W; decimalLatitude: -62.653500; decimalLongitude: -61.145723; **Identification:** identificationID: *Galerinamarginata*; identifiedBy: Fernando Augusto Bertazzo-Silva, Jair Putzke; dateIdentified: 2024; **Event:** year: 2023; month: 01; day: 24; habitat: Growing in a coastal moss field.; fieldNotes: Basidiomata are scattered in an area with vegetation predominantly composed of *Sanioniauncinata* (Hedw.) Loeske; **Record Level:** institutionCode: HBEI127; collectionCode: Fungi; basisOfRecord: PreservedSpecimen

#### Description

**Figure 2.**
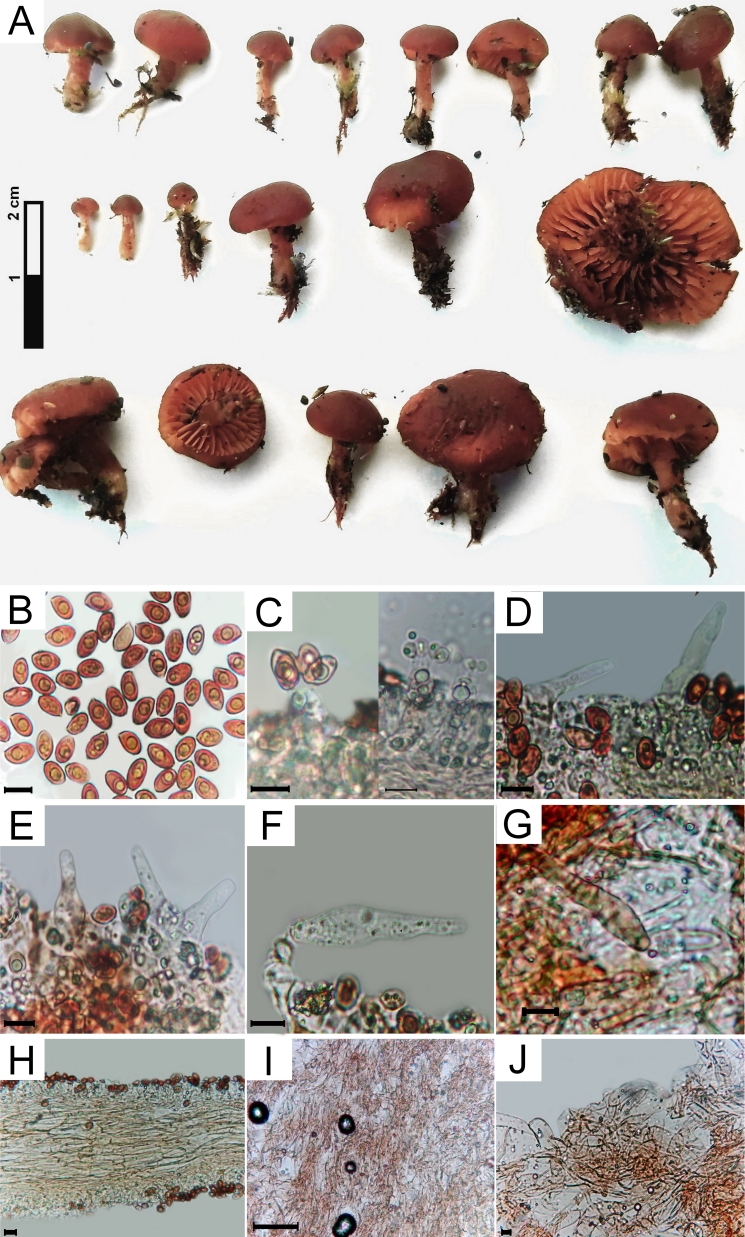
*Galerinamarginata*. **A** Basidiome in Laboratory; **B** Spores; **C** Basidia; **D** Pleurocystidia; **E, F** Cheilocystidia; **G** Caulocystidia; **H** Lamellar trama; **I, J** Pileipellis. Scale bar in B, C, D, E, F, G, H and J - 10 μm. In I - 100 μm.

Pileus 7–31 mm diameter, initially campanulate to hemispherical and then flat-convex to convex (Fig. 2A). Margin involute to revolute as the basidiome develops. Hymenophore with lamellae adnate to slightly emarginate. Stipe 2–8 x 4–17 mm, central, cylindrical. Basal mycelium is absent. Spore print not obtained (Fig. 2A). Basidiospores (9.3) 11.4 ± 1.4 (13.3) x (5.5) 6.4 ± 0.4 (7.2) µm, Q = (1.29) 1.78 (2.42), ellipsoid to amygdaliform, moderately rugulose to verrucose, heavily pigmented (Fig. 2B). Basidia (24.0) 29,3 ± 2.6 (34.3) x (6.9) 9.3 ± 1.6 (12.82) x (3.6) 5.8 ± 1.2 (8.47) µm, clavate, hyaline, four-spored (Fig. 2C). Pleurocystidia (18.4) 34.3 ± 9 (48.8) x (7.6) 10.0 ± 2.1 (14.1) x (3.6) 6.1 ± 1.9 (9.4), utriform to slightly lageniform, hyaline (Fig. 2D). Cheilocystidia (29.0) 37.8 ± 12.6 (57.79) x (7.7) 10.5 ± 1.9 (12.2) x (4.8) 5.1 ± 0.2 (5.3) x (5.2) 5.8 ± 0.4 (6.3) µm, lageniform to ventricose-fusoid and slightly fusiform, hyaline (Fig. 2E, F). Caulocystidia (59) 63.9 ± 7.9 (78.1) x (11.2) 12.7 ± 0.7 (13), hyphoid to utriform, hyaline to slightly pigmented (Fig. 2G). Pileocystidia were not observed. Regular lamellar context. Pileipellis is composed of prostrate to periclinal hyphae, encrusted with pigments (Fig. 2I, J). Clamp connections were not observed.

#### Ecology

Growing in a coastal moss field. Basidiomata are scattered in an area with vegetation predominantly composed of *Sanioniauncinata* (Hedw.) Loeske.

## Analysis

### Phylogenetic analysis

**Figure 3.**
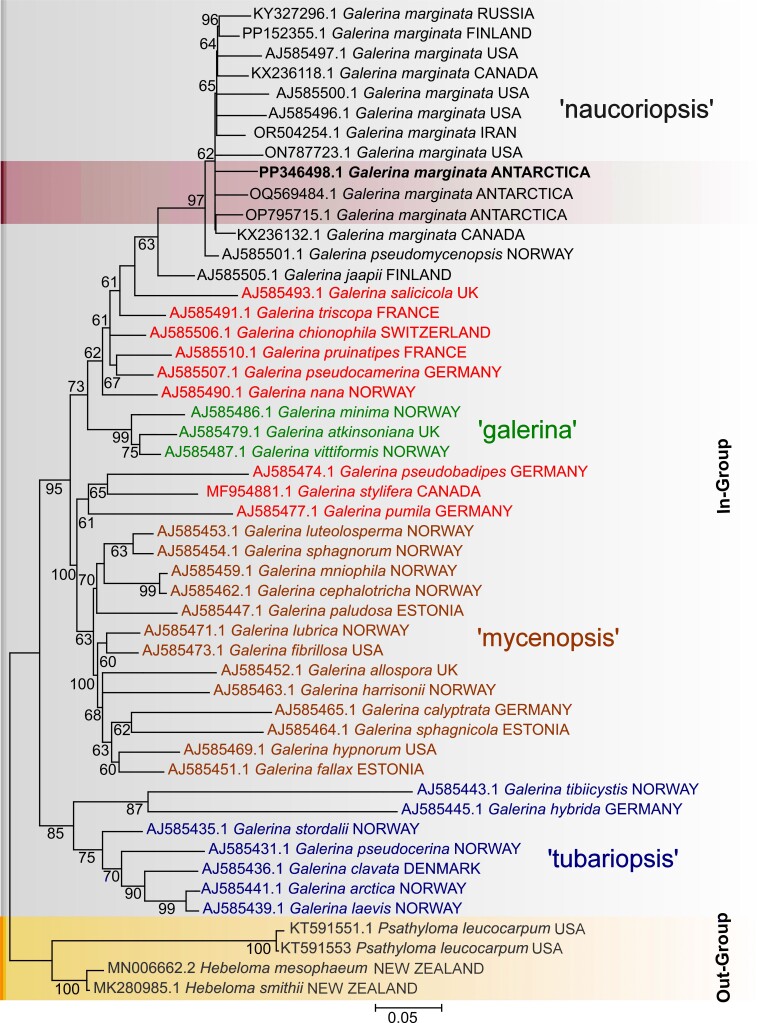
Maximum Likelihood phylogenetic tree, based on 1,303 nucleotide positions within the ITS1-5.8S-ITS2 region. The specimen isolated in this study is highlighted in bold. Antarctic specimens are highlighted in a red box. The yellow box highlights the out-group and the grey box represents the in-group. The *Galerina* lineages are divided by colours according to Guldem et al (2005): *Naucoriopsis*: black. *Galerina*: green. *Mycenopsis*: brown. *Tubariopsis*: blue. Values alongside the branches indicate bootstrap support greater than 60%. The scale bar at the bottom of the topology indicates substitutions per site, with a value of 0.05.

Phylogenetic relationships reconstructed by Maximum Likelihood demonstrate that *Galerinamarginata* forms a monophyletic clade with over 60% bootstrap support in most branches (Fig. 3). The isolate in this study was found to be internal to the main cluster. Evolutionary reconstructions using the Maximum Likelihood method indicate that the branches correspond to the Antarctic isolate being an internal clade within the *marginata* group. The same species, OQ569484.1, found in Antarctica (South Shetland Islands, Livingston Island, Punta Hannah) and OP795715.1, also in Antarctica (Norsel Point, Amsler Island), exhibited behaviour as a sister clade with over 60% bootstrap support (Fig. 3).

## Discussion

The findings presented in this study mark the third documented occurrence of *Galerinamarginata* in Antarctica and, notably, it represents the first comprehensive morphological description of an individual of this species developing on the Antarctic Continent. Previously, Arenz et al. (2014) reported the presence of *G.marginata* on Amsler Island, while Garrido-Benavent et al. (2023) conducted a phylogenetic study focusing on specimens collected in Punta Hannah, Livingston Island, to understand the evolution of this species.

The morphological characteristics, particularly the size and shape of the pileus, as well as the size and form of the spores described by Garrido-Benavent et al. (2023), closely correspond to those observed in this study, indicating similarity amongst individuals of the species found on the Antarctic Continent. However, the absence of descriptions of other structures of *G.marginata* limits more comprehensive comparisons.

In comparison with collections worldwide, the specimen collected in Antarctica and detailed in this study exhibits variations and similarities in its macroscopic characteristics (Table 2). The size of the pileus in the described specimen ranged from 7 to 31 mm, while other individuals had pilei ranging from 5 to 45 mm. Across all samples, the pileus shapes displayed remarkable similarity, with the majority of individuals initially possessing campanulate to hemispherical pilei that later became convex as the fungus developed. Nevertheless, other forms such as umbonate, depressed, flattened and bell-shaped were also documented. The attachment of the lamellae to the stem varied from adnate, subdecurrent or adnexed to slightly emarginate or decurrent. Notably, our sample was the only one displaying an emarginate shape during its development. Moreover, our specimen stands out as the individual with the smallest stipe. While stipe sizes ranged from 20 to 60 mm in other specimens, our sample exhibited a size ranging from 4 to 17 mm (Cho et al. 2016, Gulden and Hallgrimsson 2000, Akata et al. 2020, Prydiuk 2020). The aforementioned differences may be influenced by the development substrate and possible extreme climate conditions that could impact the species' growth process. However, more comprehensive studies focused on the bionomics and distribution of the species on the continent are needed for more robust confirmations (Table 2).

The microscopic morphology of the specimen examined in this study reveals both similarities and differences compared to other samples described in various countries (Table 2). Our sample exhibits spores that are relatively larger than those found in other individuals, although the ellipsoidal to amygdaliform shape is consistent across all specimens. Basidia sizes vary, ranging from 17 µm to 35.3 µm (Cho et al. 2016, Prydiuk 2020), with the basidia of our specimen falling within the average size range. Notably, basidia containing two spores were not observed in our sample, despite reports of bisporic and tetrasporic basidia found in this species (Prydiuk 2020). The cheilocystidia in our sample are similar in size to the structures described by Prydiuk (2020) and Akata et al. (2020), albeit smaller compared to the maximum sizes described, which can reach up to 65 µm (Gulden and Hallgrimsson 2000). Similarly, the pleurocystidia also demonstrate smaller sizes, differing from the maximum values described in literature, which can reach up to 77.2 µm (Cho et al. 2016) (Table 2).

Another notable characteristic observed in the individual under study is also evident in other specimens of the species collected in Antarctica (Garrido-Benavent et al. 2023). While individuals from other continents display pileus colours ranging from ochre to reddish-brown (Akata et al. 2020, Prydiuk 2020), *G.marginata* developing in Antarctica exhibits pileus colours leaning towards dark red, with rare yellow tones in its composition. These data corroborate the findings of Krah et al. (2019), who observed that mushroom assemblages in colder areas, based on the assessment of mushrooms in Europe, tend to be significantly darker. The dark colouration of *G.marginata* in the Antarctic Continent may play a determining role in its growth and development, as it can regulate temperature and, thus, contribute to its survival. Furthermore, pigmented fungi tolerate high UV radiation better than non-pigmented fungi due to their melanised cell wall, which serves as a radiation shield, aiding their survival in extreme conditions (Pulschen et al. 2015, Venil et al. 2020, Kreusch and Duarte 2021, Cavalcante et al. 2023).

In the phylogenetic reconstructions with *G.marginata* (isolate PP346498.1), a relationship within the monophyletic clade of the genus was observed and it behaved as a sister clade to other *G.marginata* found in gelid regions. Additionally, *G.marginata* showed the behaviour of a most derived clade, compared to the other internal clades of *Galerina* analysed. In other studies involving isolates of species collected in alpine regions of North America, close relationships were presented (Garrido-Benavent et al. 2023). For the Antarctic isolates, this grouping phenomenon is curiously repeated. However, the behaviour of isolates from the Polar Regions, which diversified relationships in this reconstruction, does not show a clearly defined internal unique clade. This suggests that the ancestry of *G.marginata* in this region occurred through more than one demographic expansion event during the Pleistocene. (Garrido-Benavent et al. 2023). This fact converges with our findings, as the isolate in this study, despite belonging to the *marginata* clade, did not form a monophyletic internal clade with the other *G.marginata* from Antarctica, with its branches collapsing only as sister clades.

**Figure 4.**
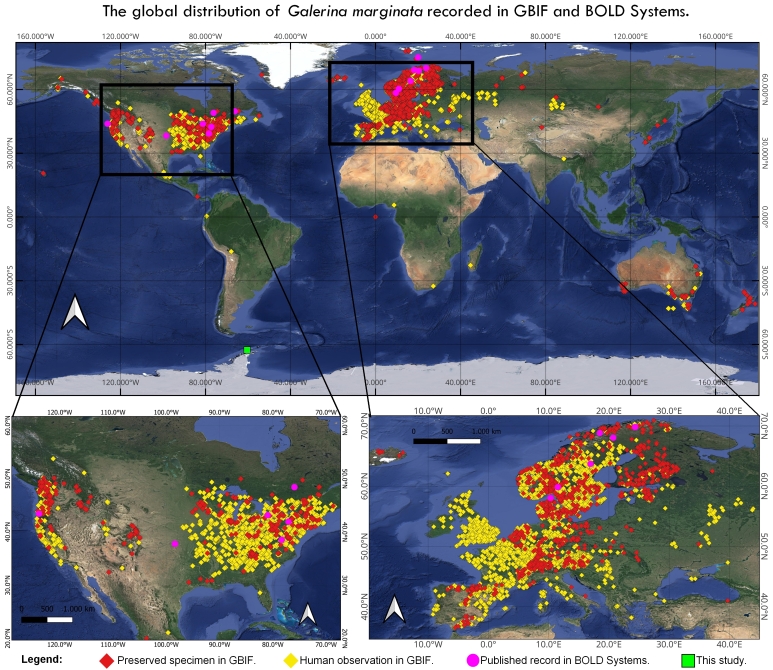
Global distribution of *Galerinamarginata*, based on records from the Global Biodiversity Information Facility (GBIF) and the Barcode of Life Data System (BOLD Systems).

The differences observed in the described specimens may reflect both environmental reactions and the development of functional traits to ensure the survival of these organisms in extreme regions. *G.marginata* exhibits a cosmopolitan distribution and further studies should be conducted to assess its bionomy and provide a comprehensive morphological description of these organisms worldwide. Searching on the Global Biodiversity Information Facility (GBIF.org 2024a) yielded 22,397 occurrences of *G.marginata* worldwide (Fig. 4). Amongst these, 80.30% represented human observations and 17.30% were occurrences with specimens preserved in herbaria and research institutions, totalling 17,678 georeferenced records (Fig. 4). Searches on the Barcode of Life Data System (Ratnasingham and Hebert 2007) resulted in 29 records of *G.marginata* worldwide, distributed across Norway, the United States and Canada (Fig. 4). Despite the significant disparity in the number of records between the systems, both indicate North America and Europe as the continents with the highest distribution of *G.marginata* globally.

However, it is important to consider that the distribution observed in the USA and Europe (Fig. 4) may be influenced by sampling effort and could reflect a bias in the number of reports. This suggests that regions with more active recording and research efforts might show higher occurrence numbers, potentially overlooking areas with less extensive biodiversity documentation. Therefore, it is crucial to conduct research focused on documenting new species records. Such efforts are essential for providing a more comprehensive understanding of global biodiversity, identifying regions of high conservation priority and ensuring that lesser-studied areas are not neglected. Expanding our understanding of biodiversity can facilitate a more accurate and comprehensive representation of species distributions worldwide.

### Conclusion

In this study, we present the third documented occurrence of *Galerinamarginata* in Antarctica, providing the first comprehensive morphological description of an individual of this species developing on the Antarctic Continent. Our findings contribute to the understanding of the geographic distribution and morphological variability of *G.marginata*, exploring its adaptations to extreme environments.

The morphological characterisation revealed both similarities and differences between the Antarctic specimen and those from other regions. While certain macroscopic characteristics, such as pileus shape, show remarkable similarity across specimens globally, variations in size and attachment of lamellae were observed. Additionally, distinctive dark colouration is observed in Antarctic specimens, which may signify an adaptation to extreme conditions, potentially assisting in temperature regulation and UV protection in the harsh polar environment. Microscopically, it differs in spore size and lacks bisporic basidia. However, fundamental traits like spore shape and basidia size remain consistent. Cheilocystidia and pleurocystidia are smaller, but still adhere to the species description.

These characteristics may be attributed to a combination of environmental and genetic factors that influence the development and morphology of fungi in the Antarctic Region. However, more comprehensive studies on the bionomics and distribution of the species on the continent are needed to corroborate these observations and better understand fungal diversity in Antarctica.

Additionally, the phylogenetic relationship observed, with Antarctic isolates forming a sister clade to other specimens from cold regions, implies a shared evolutionary history amongst cold-adapted populations of *G.marginata*. This relationship suggests the possibility of historical demographic events during the Pleistocene that influenced the dispersal and diversification of the species.

Thus, the data presented in this study serve as an initial foundation for subsequent monitoring of the *G.marginata* population in Antarctica. The analysis of the individuals described in this research enables the assessment of the potential impacts of climate change on the development and survival of these organisms in the coming years. This study not only fosters, but also points towards future research dedicated to the bionomics, phylogeny and distribution of *G.marginata* in Antarctica.

## Supplementary Material

XML Treatment for Galerina marginata

## Figures and Tables

**Table 1.** Species and GenBank accession numbers of sequences used in this study (newly-generated sequence are indicated in bold).

*S*pecies	Strain/Voucher	Location	GenBank Accession No.
*Galerinaallospora*	O 73460	United Kingdom	AJ585452.1
*Galerinaarctica*	O 50535	Norway	AJ585441.1
*Galerinaatkinsoniana*	O 73459	United Kingdom	AJ585479.1
*Galerinacalyptrata*	O 73449	Germany	AJ585465.1
*Galerinacephalotricha*	O 154146	Norway	AJ585462.1
*Galerinachionophila*	O 73463	Switzerland	AJ585506.1
*Galerinaclavata*	O 72166	Denmark	AJ585436.1
*Galerinafallax*	O 154355	Norway	AJ585451.1
*Galerinafibrillosa*	MICH 40850	USA	AJ585473.1
*Galerinaharrisonii*	O 50711	Norway	AJ585463.1
*Galerinahybrida*	O 73458-2	Germany	AJ585445.1
*Galerinahypnorum*	MICH 46302	USA	AJ585470.1
*Galerinajaapii*	O 154387	Finland	AJ585505.1
*Galerinalaevis*	O 70903	Norway	AJ585439.1
*Galerinalubrica*	O 154034	Norway	AJ585471.1
*Galerinaluteolosperma*	O 154076	Norway	AJ585453.1
*Galerinamarginata*	O 72427	USA	AJ585500.1
*Galerinamarginata*	O 300011	USA	AJ585497.1
*Galerinamarginata*	O 72507	USA	AJ585496.1
*Galerinamarginata*	UBC F32027	Canada	KX236118.1
***Galerinamarginata***	**LFGM 2401**	**Antarctica**	**PP346498.1**
*Galerinamarginata*	ARIOS_GalMar	Antarctica	OQ569484.1
*Galerinamarginata*	SAT-21-248-01	USA	ON787723.1
*Galerinamarginata*	Amsler-2012	Antarctica	OP795715.1
*Galerinamarginata*	ZR4	Iran	OR504254.1
*Galerinamarginata*	UBC F32036	Canada	KX236132.1
*Galerinamarginata*	191	Finland	PP152355.1
*Galerinamarginata*	LE-BIN 2479	Russia	KY327296.1
*Galerinaminima*	O 154480	Norway	AJ585486.1
*Galerinamniophila*	O 60574	Norway	AJ585459.1
*Galerinanana*	O 153723	Norway	AJ585490.1
*Galerinapaludosa*	O 73462	Estonia	AJ585447.1
*Galerinapruinatipes*	O 73438	France	AJ585510.1
*Galerinapseudobadipes*	O 154252	Norway	AJ585474.1
*Galerinapseudocamerina*	O 73471	Germany	AJ585507.1
*Galerinapseudocerina*	O 153998	Norway	AJ585431.1
*Galerinapseudomycenopsis*	O 50526	Norway	AJ585501.1
*Galerinapumila*	O 73440	Germany	AJ585477.1
*Galerinasalicicola*	K 99448	United Kingdom	AJ585493.1
*Galerinasphagnicola*	O 73441	Estonia	AJ585464.1
*Galerinasphagnorum*	O 70913	Norway	AJ585454.1
*Galerinastordalii*	O 154169	Norway	AJ585435.1
*Galerinastylifera*	UBC F-25666	Canada	MF954881.1
*Galerinatibiicystis*	O 72930	Norway	AJ585443.1
*Galerinatriscopa*	O 73453	France	AJ585491.1
*Galerinavittiformis*	O 154565	Norway	AJ585487.1
*Hebelomamesophaeum*	NYS:NYS-F-001411	USA	MN006662.2
*Hebelomasmithii*	MICH:MICH 10730	USA	MK280985.1
*Psathylomaleucocarpum*	JAC12071	New Zealand	KT591551.1
*Psathylomaleucocarpum*	K. Soop KS-BR185	New Zealand	KT591553.1

**Table 2.** Morphological characterisation of *Galerinamarginata*.

	**This study**	**Gulden and Hallgrimsson (2000)**	**Cho et al. (2016)**	**Prydiuk (2020)**	**Akata et al. (2020)**
	Antartica	Iceland	Korea	Ukraine	Turkey
**Pileus**	7–31 mm, initially campanulate to hemispherical and then flat-convex to convex	10-40 mm, conic, ± umbonate or even papillate, becoming convex to plane, sometimes also slightly depressed, moist ± sticky to viscid	15–35 mm, convex or conical when young, then expanded, piano-convex or flattened when mature	5-30 mm, initially hemispherical, bell-shaped or convex, later convex-spreading to spreading, often with a low bump in the centre	20–45 mm, broad, hemispherical at first and then became convex to plano-convex with an obtuse umbo
**Lamellae**	Adnate to slightly emarginate	Adnate to slightly decurrent	Subdecurrent	Adnate	Adnexed to slightly decurrent
**Stipe**	4–17 x 2–8 mm	20–60 x 2–5 mm	20–40 × 4–8 mm	20–55 mm x 2–5 mm	22–40 mm × 2–5 mm
**Spore**	(9.3) 11.4 (13.3) x (5.5) 6.4 (7.2) µm, Q = (1.29) 1.78 (2.42), ellipsoid to amygdaliform, moderately rugulose to verrucose	(8.5-)9–10.5 x 5.5–6.5 μm, Av (40/4) = 9.7 x 5.9 μm, Q = 1.5-1.8, Q./30) = 1.6, amygdaliform to ellipsoid, rugulose-verruculose	(9.4) 9.7 (10.2) × (5.6) 6.1 (6.7) μm, Q = (1.47) 1.60 (1.72), ellipsoidal to oval	(7,5–)8,0–9.5(–11.0) × 5.0–6.5 μm, Q = 1,36– 1.83; av. L = 8.9 ± 0.71 μm, av. B = 5.7 ± 0.37 μm, av. Q = 1.57 ± 0.09, rugulose to verrucose	8–10 × 5–6 μm, elliptical to amygdaliform, moderately verrucose
**Basidia**	(24.0) 29.3 (34.3) x (6.9) 9.3 (12.82) x (3.6) 5.8 (8.47), clavate, four-spored	22.5–32 x 7.5–8 μm, constricted, four-spored	27.7–35.3 × 7.8–10.4 μm, clavate, four-spored	17.0–25.0 × 7.0–8.5 μm, club-shaped, two- and four-spored	25–30 × 7–8 μm, cylindrical to clavate, hyaline, four-spored
**Cheilocystidia**	(29.0) 37.8 (57.79) x (7.7) 10.5 (12.2) x (4.8) 5.1 (5.3) x (5.2) 5.8 (6.3) µm, lageniform to ventricose-fusoid	40–65 x 9–14.5 x 3–5 x 3–7 μm, ventricose- (sub)capitate, head often ellipsoid to spearhead-shaped, rarely tip not inflated	51.3–62.2 × 8.2–9.1 μm, fusiform-ventricose to obclavate, abundant	35.0–50.0 × 7.0–14.5 μm, spindle-shaped, apex rounded or slightly thickened	35–55 × 10–15 μm, lageniform to fusiform
**Pleurocystidia**	(18.4) 34.3 (48.8) x (7.6) 10.0 (14.1) x (3.6) 6.1 (9.4), Utriform to slightly lageniform	Scattered, often few, similar to cheilocystidia	52.4–77.2 × 11.4–13.9 μm, fusiform-ventricose to obclavate	40.0–75.0 × 13.0–17.0 μm, spindle-shaped, apex rounded or slightly thickened	Similar to cheilocystidia
**Caulocystidia**	(59) 63.9 (78.1) x (11.2) 12.7 (13), hyphoid to utriform, hyaline to slightly pigmented	Rather few, similar to the cheilocystidia	-	Two types: a) 50.0–85.0 × 9.5–17.0 μm, spindle-shaped, apex rounded or slightly thickened; b)20.0–31.0 × 6.5–8.0 μm, club-shaped	-
**Pileipellis**	Prostrate to periclinal hyphae, encrusted with pigments	-	-	Hyphae 2.5–6.0 μm thick, somewhat mucilaginous in the upper layers, with a light granular pigment encrustation	Periclinal hyphae, hyaline to light brownish, 3–5 broad and had some septa with clamps
